# Strong and Elastic Hydrogels from Dual-Crosslinked Composites Composed of Glycol Chitosan and Amino-Functionalized Bioactive Glass Nanoparticles

**DOI:** 10.3390/nano12111874

**Published:** 2022-05-30

**Authors:** Qing Min, Congcong Wang, Yuchen Zhang, Danlei Tian, Ying Wan, Jiliang Wu

**Affiliations:** 1 School of Pharmacy, Hubei University of Science and Technology, Xianning 437100, China; baimin0628@hbust.edu.cn (Q.M.); zhangych@hbust.edu.cn (Y.Z.); 2 College of Life Science and Technology, Huazhong University of Science and Technology, Wuhan 430074, China; congcongwang@hust.edu.cn (C.W.); tiandanlei@hust.edu.cn (D.T.)

**Keywords:** dual-crosslinked hydrogel, glycol chitosan, bioactive glass nanoparticles, amino functionalization, strength and elasticity

## Abstract

Mesoporous bioactive glass (BG) nanoparticles (NPs) with a high specific surface area were prepared. The surfaces of BG NPs were further modified using an amino-containing compound or synthesized precursors to produce three kinds of amino-functionalized bioactive glass (ABG) NPs via devised synthetic routes. The achieved ABG NPs possessed various spacer lengths with free amino groups anchored at the end of the spacer. These ABG NPs were then combined with glycol chitosan (GCH) to construct single- or dual-crosslinked ABG/GCH composite hydrogels using genipin (GN) alone as a single crosslinker or a combination of GN and poly(ethylene glycol) diglycidyl ether (PEGDE) as dual crosslinkers. The spacer length of ABG NPs was found to impose significant effects on the strength and elasticity of GN-crosslinked ABG/GCH hydrogels. After being dually crosslinked with GN and PEGDE, the elastic modulus of some dual-crosslinked ABG/GCH hydrogels reached around 6.9 kPa or higher with their yielding strains larger than 60%, indicative of their strong and elastic features. The optimally achieved ABG/GCH hydrogels were injectable with tunable gelation time, and also able to support the growth of seeded MC3T3-E1 cells and specific matrix deposition. These results suggest that the dual-crosslinked ABG/GCH hydrogels have the potential for some applications in tissue engineering.

## 1. Introduction

Injectable hydrogels have drawn a lot of attention in tissue engineering applications over the last decade due to their several advantages, such as a minimally invasive injection procedure, the in situ formation of self-supporting objects and convenient filling of complex tissue defects with arbitrary shapes [1,2]. In addition to these, they can serve as injectable carriers for delivering cells, therapeutic drugs and bioactive molecules because they have highly interconnected porous structures with a large amount of water retention as well as good permeability, which is particularly conducive to the easy transport of nutrients and metabolites, and, in turn, beneficial for the cell-involved tissue repair and reconstruction [2,3,4]. Nowadays, many kinds of naturally sourced polymers, majorly including collagen, gelatin, silk fibroin, chitosan (CH), dextran, alginate and hyaluronic acid, have been extensively used in the form of hydrogels since they usually show good biocompatibility, easy biodegradation and better biomedical performance than other types of hydrogels made from synthetic polymers [1,2,3,4,5,6,7].

Among the mentioned natural polymers, CH is recognized as a versatile biomaterial and has been broadly investigated for hydrogel applications due to its meritorious properties, including biodegradability, nontoxicity, nonantigenicity, anti-microbial activity, adherence, cell affinity and a broad range of abilities for chemical modification through its amino at the C-2 sites or hydroxyl groups at the C-6 sites [8,9]. In particular, CH molecules have a chemical structure highly similar to glycosaminoglycans (GAGs) that commonly exist in the extracellular matrix (ECM) of different types of human tissues. These advantages of CH have made it a preferable biomaterial for a large variety of applications in tissue repair and regeneration [8,10]. To date, many types of CH-based hydrogels have been developed via various physical or chemical methods and they have been widely utilized for the repair and regeneration of injured skin, nerve, cartilage and bone [8,9,11]. Despite the wide-ranging usability, the hydrogels based on innate CH often show poor mechanical properties such as fragility and insufficient strength, limiting their applications to some extent.

In the case of bone repair and regeneration, the applicable hydrogels need to have the ability to build a strong and elastic microenvironment for housing cells because the mechanical properties of the hydrogels applied can markedly modulate the growth behavior of the cells encapsulated in or migrating from the periphery of injured tissue and, concomitantly, exert a strong effect on the remodeling of the neonatal tissues. Nowadays, it is generally recognized that a mechanically poor hydrogel would usually result in inferior outcomes if it is used in the repair and regeneration of some skeletal tissues, such as bone or articular cartilage [12,13]. Accordingly, how to endow a hydrogel with high strength and good elasticity as well as sufficient safety is always an important issue that needs to be addressed if it is intended for use in bone repair.

A large number of studies have suggested that a dual or multiple network polymer hydrogel could be substantially strengthened in terms of its three-dimensional (3D) stability, mechanical performance and degradation tolerance through interactions such as the mutual restriction of networks, chain entanglement and intermolecular interactions when compared to a single network gel [14,15,16]. Hence, it can be envisaged that a strong and elastic CH-based composite hydrogel with the potential for use in bone repair could be developed, provided that some other suitable components are employed while the resulting composite hydrogel is effectively crosslinked using suitable crosslinkers. In addition to the employment of strong and elastic hydrogels for housing cells in bone repair, the incorporation of certain osteogenic ingredients into the gels is one of the practicable strategies to promote bone repair [3,17]. Various kinds of inorganic biomaterials such as hydroxyapatite, beta-tricalcium phosphate and bioactive glass (BG) have been widely investigated for use in bone repair. Of them, BG nanoparticles (NPs) have two specific advantages in bone repair: (1) they can firmly bond to bone tissue in the defect and transform into hydroxyapatite-like layers under the action of physiological fluids [3,17,18], and (2) their bioactive dissolution products such as Si and Ca ions have the capability for osteogenic stimulation [17,18]. Nevertheless, it is generally difficult to build a strong and elastic composite hydrogel by simply mixing CH and BG NPs while crosslinking the resulting mixture because BG NPs only contain hydroxyl groups on their surface and these hydroxyl groups are difficult to use for crosslinking with CH. Hence, new strategies for effectively crosslinking BG NPs with CH need to be explored for constructing strong and elastic BG/CH composite hydrogels if the resulting gels are intended for use in bone repair.

With these considerations in mind, in this study, an effort was made to develop a new type of injectable composite hydrogel using glycol chitosan (GCH), a water-soluble derivative of CH, and amino-functionalized bioactive glass (ABG) NPs while employing genipin (GN) as a single crosslinker or a combination of GN and poly(ethylene glycol) diglycidyl ether (PEGDE) as dual crosslinkers. Mesoporous BG NPs with a high specific surface area were first prepared, and their surfaces were modified using an amino-containing compound or synthesized precursors to produce three kinds of ABG NPs via newly designed synthetic routes. These ABG NPs were endowed with different spacers with varying spacer lengths and the free amino groups were anchored at the end of the spacer. ABG NPs were then combined with GCH to construct single or dual-crosslinked ABG/GCH composite hydrogels. GN, a natural product that is derived from the fruits of the *Gardenia jasminoides* plant, was selected as an amino-associated crosslinker due to its much higher safety when compared to many other kinds of crosslinkers suited for crosslinking amino groups [8,19,20]. PEGDE was employed as another crosslinker considering that its epoxy groups have reactivity toward amino and hydroxyl groups while showing much faster reaction rates compared to GN. Some optimally constructed ABG/GCH gels were found to exhibit mechanically strong and elastic characteristics with well-defined injectability and tunable gelation time. They were able to support the growth of seeded MC3T3-E1 cells and specific matrix deposition. All these results suggest that the presently developed ABG/GCH composite gels could find a new avenue for potential application in bone tissue engineering.

## 2. Materials and Methods

### 2.1. Materials

GCH (degree of polymerization ≥ 400), GN and PEGDE (M_n_ of PEG: 2000) were purchased from Sigma-Aldrich (Shanghai, China). Cetyltrimethylammonium bromide (CTAB), tetraethyl orthosilicate (TEOS), 3-(aminopropyl)triethoxysilane (APTES), diethylenetriamine (DETA), 3-chloropropyldimethylmethoxysilane (CPDMMS) and pentaethylenehexamine (PEHA) were supplied by Aladdin Inc. (Shanghai, China). Other chemicals were of analytical grade and purchased from Sinopharm (Shanghai, China).

### 2.2. Synthesis of Bioglass Nanoparticles

BG NPs were synthesized following a reported method with some modification [21]. In a typical process, 0.5 g of CTAB was dissolved in a mixed solution composed of 70 mL of deionized water, 0.8 mL of aqueous ammonia, 5 mL of ethyl ether, and 10 mL of ethanol, and the resulting mixture was vigorously stirred for 30 min. To this mixture, 2.5 mL of TEOS was added with additional stirring for 30 min, followed by the addition of 0.47 g of calcium nitrate (Ca(NO_3_)_2_⋅4H_2_O). The mixture was allowed to react with stirring for 6 h at room temperature. The resulting sediment was collected by centrifugation at 10,000 rpm for 5 min, washed with ethanol three times first, and then with deionized water repeatedly. The obtained BG NPs were dried at 70 °C for 24 h. The ground powder was heated up to 550 °C at a rate of 2 °C/min to remove organic residues. CaO/SiO_2_ molar ratio for the prepared BG NPs was measured to be around 0.14 from their energy dispersive spectra.

### 2.3. Amino Functionalization of Bioglass Nanoparticles

**Figure 1:**
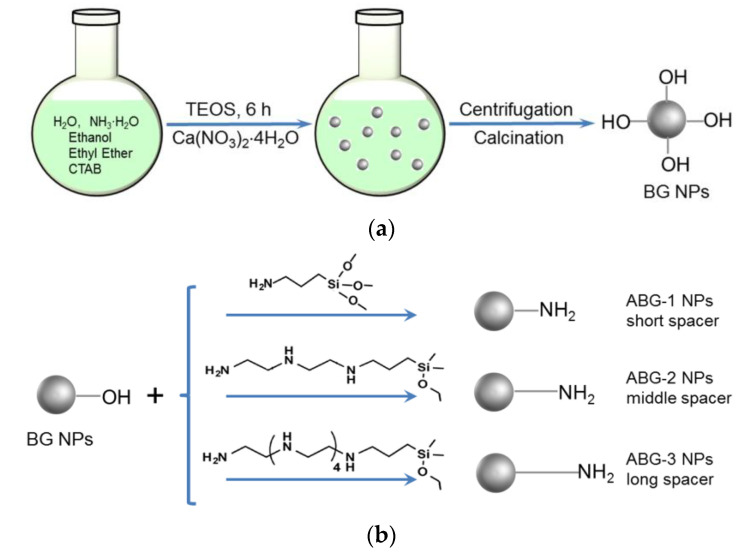
Scheme for synthesis of BG NPs (**a**) and different kinds of ABG NPs (**b**) with varied spacer lengths.

Three kinds of ABG NPs were prepared via different synthetic routes. BG NPs were directly reacted with APTES in dry toluene to produce a kind of ABG NP (denoted ABG-1 NPs) using a method similar to that described in the literature [22]. Two other kinds of ABG NPs were prepared as follows. In brief, a given amount of DETA was dissolved in 5 mL of anhydrous ethanol, in presence of CPDMMS and triethylamine, with stirring for 1 h to produce a precursor. After that, a suspension of BG NPs in ethanol (0.2 wt%) was added to the system containing the precursor with stirring at reflux for 24 h. The product was recovered by centrifugation, washed with anhydrous ethanol repeatedly and dried at 120 °C to achieve the second kind of ABG NP (denoted ABG-2 NPs). The third kind of ABG NP, denoted ABG-3 NPs, was prepared using the same method except that DETA was replaced by PEHA. A schematic illustration for the synthesis of BG NPs and three kinds of ABG NPs with varied spacer lengths is presented in Figure 1. The feed ratios of BG NPs to the amino-containing compound or other compounds were optimized using the orthogonal design method to ensure that three kinds of ABG NPs had similar amino amounts but varied spacer lengths. The amino content of these ABG NPs was detected by the ninhydrin assay [23].

### 2.4. Characterization

NPs were viewed with a transmission electron microscope (TEM, Tecnai, FEI, Hillsboro, OR, USA) to identify their morphology, size and dispersity. A dynamic light scattering instrument (Nano-ZS90, Malvern, Worcestershire, UK) was used to detect the hydrodynamic size and zeta (ζ) potential of NPs. Energy dispersive X-ray (EDX) spectra of NPs were detected during scanning electron microscopy (SEM, Quanta, FEI, Eindhoven, The Netherlands). For the measurements of isotherms and pore-size distributions, NPs were first dried in a vacuum oven at 100 °C for 12 h before loading into the sample chamber of the surface area and pore size analyzer (ASAP 2020 Plus, Micromeritics, Norcross, GA, USA). After being degassed at 120 °C for 24 h, the volume of nitrogen adsorption–desorption was measured at different pressures. The specific surface areas of NPs were determined using the BET method, and their pore size and volume were calculated with the BJH method.

### 2.5. Preparation of Hydrogels

GCH solutions with varying concentrations were prepared by dissolving GCH in deionized water. Three kinds of ABG NPs were dispersed in deionized water to produce their respective suspensions. A series of composite solutions with formulated compositions were prepared by mixing the selected GCH solution, ABG suspension and GN solution together. The prepared composite solutions were then introduced into different vials, and incubated at 37 °C for 12 h to produce the single-crosslinked gels.

In the case of dual-crosslinked ABG/GCH gel preparation, PEGDE was added to the above prepared composite solutions to serve as another crosslinker. The obtained composite solutions were also introduced into different vials and the vials were incubated at 37 °C for varied periods. Gelation time for dual-crosslinked gels was determined using a tube-inverting method. In a typical process, one of the preparatory composite solutions was introduced into a vial, and the vial was placed in an ice/water bath with stirring for 5 min before incubation. Once the incubation of the vial in the water bath (37 °C) began, the fluidity of the solution in the vial was regularly checked, and gelation time was recorded starting from the time point for the vial incubation and ending at the moment when the solution stopped flowing.

### 2.6. Rheological Measurements

A rheometer (Kinexus Pro KNX2100, Southborough, MA, USA) was used for rheological measurements. Frequency sweep spectra for elastic modulus (G′) and viscous modulus (G″) of gel samples were detected in a frequency range between 0.1 and 100 Hz at 37 °C and a constant strain of 1%. Concerning strain sweep spectra, G′ and G″ of gels were detected by setting the temperature at 37 °C and frequency at 1 Hz, respectively. Shear viscosity measurement was conducted in a shear rate range between 0.1 and 100 s^−1^ at 25 °C using liquid samples.

### 2.7. Cell Culture

An osteoblast-like cell line (MC3T3-E1) was procured from the Type Culture Collection of the Chinese Academy of Sciences and used to evaluate the gel potential in supporting the cell growth. The purchased cells were expanded by culturing them in the α-MEM medium that contains fetal bovine serum (10%), penicillin (1%) and streptomycin (1%) in a 5% CO_2_ humidified atmosphere at 37 °C with medium changes every two days. The harvested cells were resuspended in PBS and used for subsequent experiments.

Cell proliferation was assessed by measuring the DNA content in cell–gel constructs. In a typical procedure, the selected composite solutions were sterilized by introducing them into different glass dishes to form their respective thin layers, and these dishes were irradiated by UV light at 4 °C for a required period of time. Afterward, an aliquot of the sterilized solution was placed in a sterile petri dish and mixed with a given volume of medium containing MC3T3-E1 cells to produce a mixture. The cell-containing mixture was loaded into 24-well culture dishes at a designated volume of 200 μL per well and incubated at 37 °C for gelling. The formed gels were then cultured with a complete medium for 7 days with medium changes every 2 days. At predetermined time intervals, gel samples were withdrawn, washed with PBS and crushed into powder in liquid nitrogen. The powder samples were then cultured with a solution containing proteinase K at 55 °C for 48 h to digest proteins in the powder samples. The supernatant matching with each sample was collected by centrifugation, and then subjected to DNA content determination using a Quant-iT PicoGreen dsDNA kit (Invitrogen) following the manufacturer’s instructions. Two-dimensional cell culture was used as a control (named 2D-control) [24].

The selected cell–gel constructs were detected using a live/dead staining method to examine the viability of the seeded cells. Typically, the above prepared cell-containing mixture was loaded into confocal dishes and incubated at 37 °C for gelling. After 3 and 7 days of incubation, the cell–gel constructs were washed with PBS and then cultured with a serum-free medium containing calcein acetoxymethyl ester and propidium iodide in the dark for cell staining. After washing with PBS, the gels were immediately imaged using a confocal microscope (LSM 510 META, Zeiss, Shanghai, China).

The activity of alkaline phosphatase (ALP) of the seeded cells and the amount of cell-synthesized type-I collagen were quantitatively measured, respectively. The above prepared cell–gel constructs were cultured in the complete medium for various durations up to 14 days, and at prescribed time points, they were washed with PBS, crushed and lysed in the lysis buffer at 4 °C. The collected supernatants were then assayed with an ALP kit (Beyotime, Shanghai, China) and a collagen type I ELISA kit (Biological, Salem, MA, USA), respectively. The amount of total protein content in cell–gel constructs was also assayed with a bicinchoninic acid protein kit (Beyotime, China).

### 2.8. Statistical Analysis

Data were shown as means ± standard deviation. Statistical difference between two groups was determined using Student’s *t*-test, and multiple comparisons were made using one-way analysis of variance. A *p*-value *<* 0.05 was considered statistically significant.

## 3. Results and Discussion

### 3.1. Characterization of Bioglass Nanoparticles

**Figure 2:**
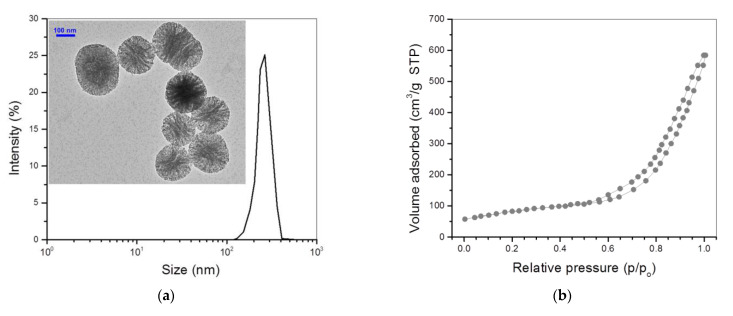

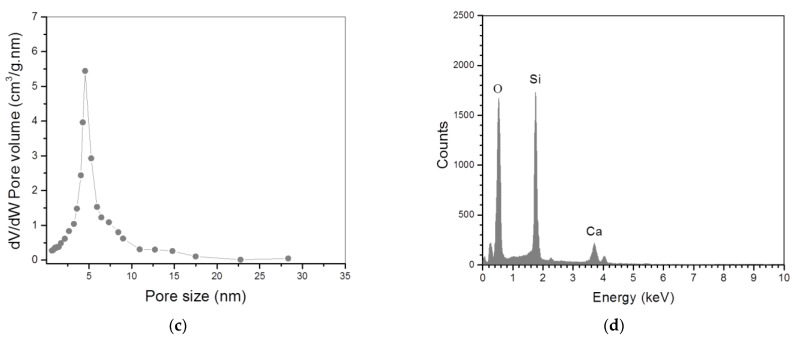
Size distribution with inserted TEM micrograph (**a**), nitrogen adsorption–desorption isotherm (**b**), pore-size distribution (**c**) and EDX spectrum (**d**) for BG NPs.

BG NPs with a high specific surface area were first synthesized under optimized conditions, intending to achieve higher surface amino substitution in subsequent chemical modification. The inserted TEM image in Figure 2a shows that BG NPs were approximately spherical with highly porous morphology. Their size varied from around 140 to 400 nm and exhibited a nearly symmetrical size distribution. The recorded N_2_ adsorption–desorption isotherm for BG NPs (Figure 2b) signifies that they had a typical hysteresis loop with the inception turning point of around 0.57 (p/p_0_), indicative of the presence of mesoporous pores inside these BG NPs [25]. The pore-size distribution in Figure 2c exhibits that most of the pores in BG NPs were about 5 nm in size, and a small percentage of pores had a size notably larger than 5 nm. Oxygen, silicon and calcium elements were detected from the EDX spectrum of BG NPs (Figure 2d). Based on the data shown in Figure 2d, the presently synthesized BG NPs were estimated to contain 88 mol% SiO_2_ and 12 mol% CaO, respectively. Several sets of BG NPs were measured to determine their specific surface area, pore volume, pore size, ζ-potential and hydrodynamic size; relevant parameters are provided in Table 1.

Three kinds of ABG NPs were produced through different synthetic routes. ABG-1 NPs were prepared via a condensation reaction between ethoxy groups in APTES and hydroxyl groups on the surface of BG NPs. Thus, free amino groups of APTES were exposed on the surface of ABG-1 NPs and connected to Si atoms by the spacer containing four chain units, as shown in Figure 1. With respect to ABG-2 and ABG-3 NPs, they were prepared by reacting BG NPs with different precursors. One of the precursors was synthesized via the reaction between CPDMMS and DETA, and the other was obtained by reacting CPDMMS with PEHA. Based on the presently designed synthetic routes, ABG-2 and ABG-3 NPs had notably longer spacer lengths in comparison to ABG-1 NPs. The spacer for ABG-2 NPs consisted of 10 chain units from the free terminal amino group to the surface Si atom of ABG-2 NPs, and, correspondingly, the spacer for ABG-3 NPs contained 19 chain units, as illustrated in Figure 1. Theoretically, it seems that the precursor with longer spacers can be synthesized following the current method. Our tentative preliminary experiments showed that if a diamino-terminal linear molecule with a longer chain length was used to synthesize the intended precursor, the obtained product would likely contain more by-products and these by-products were difficult to remove. Therefore, the spacer length for these ABG NPs was controlled at 19 chain units or lower.

**Figure 3:**
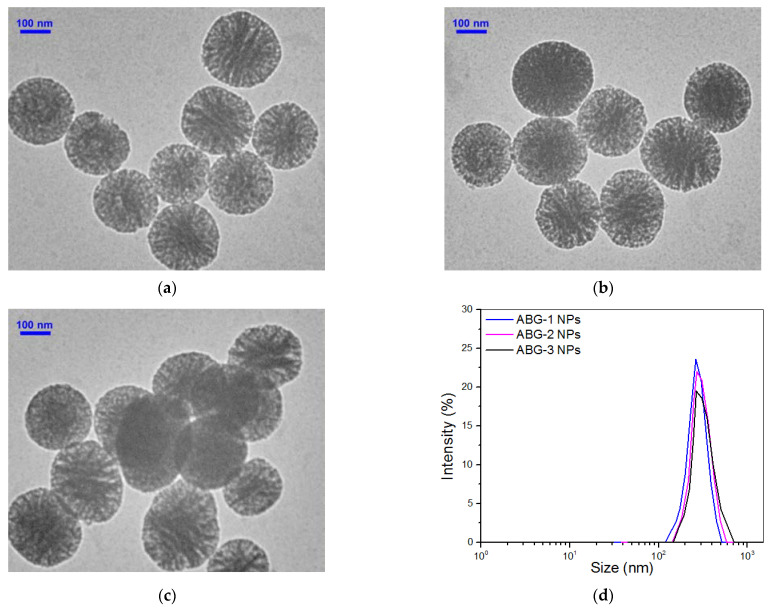
TEM micrographs for ABG-1 (**a**), ABG-2 (**b**) and ABG-3 (**c**) NPs as well as their size distributions (**d**).

Several representative TEM micrographs for three kinds of ABG NPs are shown in Figure 3. These ABG NPs were still approximately spherical with porous morphologies and well-defined dispersity; their size distribution intervals became wider but the distribution curves showed better symmetry in comparison to BG NPs. Many sets of specimens for each kind of ABG NP were subject to the assigned measurements, and obtained parameters for them are also summarized in Table 1. It can be noticed that these ABG NPs had a smaller specific surface area, pore volume and pore size but a larger particle size, and, in particular, positive ζ-potential when compared to BG NPs. As described in the experimental section, ABG NPs were produced by modifying BG NPs with an amino-containing compound or two kinds of precursors. Since BG NPs are highly porous with a hydroxyl group-exposed surface, the compound or precursors employed would thus react with the hydroxyl groups on the surface of pores inside the BG NPs in addition to their reaction with the hydroxyl groups on the surface of BG NPs, which would thus lead to decreases in the specific surface area, pore volume and pore size for ABG NPs. The three kinds of ABG NPs had similar ζ-potentials, indirectly suggesting that the amount of amino groups exposed on their surfaces would be similar. Quantitative measurements further confirmed that their surface contained about 0.4 mmol/g amino groups without significant difference (Table 1).

It is known that the hydroxyl group-exposed surface of BG NPs generally makes their ζ-potential negative. After modifying the BG NPs with the selected compound and synthesized precursors with free amino groups situated at the end of their spacers, these free amino groups will thus be exposed on the surface of ABG NPs, and, meanwhile, these amino groups are connected to the NPs by different spacers with varying lengths (Figure 1). As a result, ABG NPs attain positive ζ-potential and an enlarged hydrodynamic size compared to BG NPs.

### 3.2. Single-Crosslinked Hydrogels

**Figure 4:**
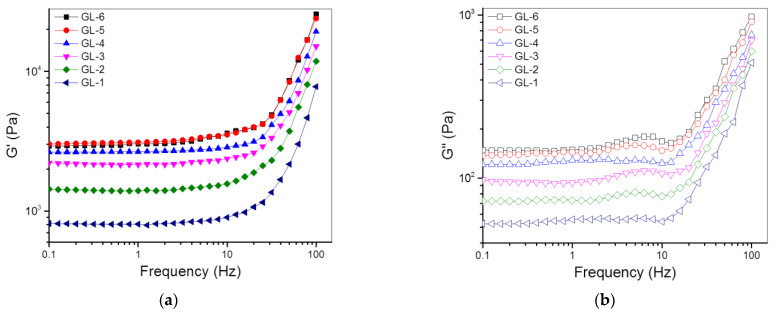

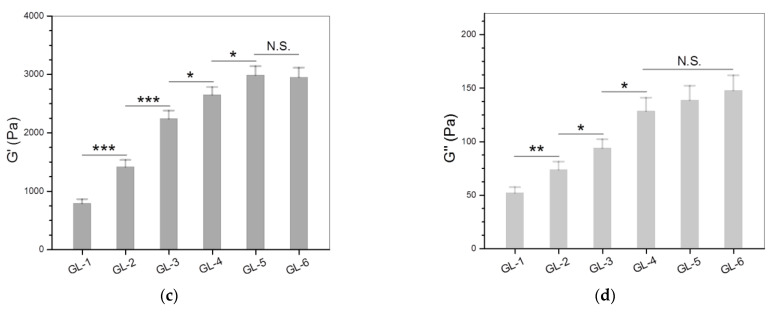
Frequency-dependent variations in G′ (**a**) and G″ (**b**) as well as average values of G′ (**c**) and G″ (**d**) at 1 Hz for gels illustrated in Table 2 (*, *p* < 0.05; **, *p* < 0.01; ***, *p* < 0.001; N.S., not significant).

Three kinds of ABG NPs were combined with GCH to prepare single-crosslinked ABG/GCH composite gels using GN only as the crosslinker. The compositional proportions and frequency sweep spectra for the resulting gels are provided in Table 2 and Figure 4, respectively. The applied amount of GN was already preset to a level of no more than 0.2% for effectively crosslinking these gels while endowing them with sufficient safety. The frequency sweep spectrum of the GL-1 gel in Figure 4a exhibits that the G′ value in the linear viscoelastic region (LVR) was around 800 Pa, meaning that the GL-1 gel is mechanically weak [26]. GL-2, GL-3 and G-4 gels that were incorporated with varied amounts of ABG-1 NPs showed much higher G′ compared to the GL-1 gel, and the G′ values of these gels in the LVR remarkably increased with an increasing amount of ABG-1 NPs. Although an amount of ABG-1 NPs higher than 2 (*w*/*v*)% can also be incorporated into these gels, the resulting composite solutions were found to be inconducive for injectable applications due to their increased viscosity. Accordingly, the amount of ABG-1 NPs incorporated was controlled at 2 (*w*/*v*)% or lower. As shown in Table 2, GL-4, GL-5 and G-6 gels were built by combining GCH with different kinds of ABG NPs having various spacer lengths, and their frequency sweep spectra exhibit that these gels had their G′ values in the LVR much higher than those of others. To quantitatively compare the gels enumerated in Table 2, many sets of gel samples were measured to determine their G′ and G″ at 1 Hz, and the relevant results are depicted in Figure 4c,d. In principle, the magnitude of G′ and the G′/G″ ratio can be used together to assess the strength of the hydrogel [26,27]. In general, a mechanically strong hydrogel has large G′, and, conjointly, its G′ is one order or even two orders of magnitude greater than its G″ [26,28,29]. The bar graphs in Figure 4c,d explicate that GL-*i* (*i* = 2, 3, 4, 5 and 6) gels had their respective G′ values of about 1.4, 2.2, 2.6, 2.9 and 2.9 kPa, respectively, with matched G′/G″ ratios of around 19.3, 23.1, 20.7, 21.6 and 20.6, respectively. Among these gels, GL-3, GL-4, GL-5 and GL-6 gels can be considered to have mechanically strong characteristics because their G′ values are larger than 2 kPa and the corresponding G′/G″ ratio is higher than 20. It can also be noticed that GL-5 and GL-6 gels showed significantly larger G′ compared to GL-4 gel. Taking into consideration the difference in ABG NPs employed for producing GL-4, GL-5 and GL-6 gels (Table 1 and Table 2), it can be concluded that the ABG NPs with a longer spacer length can serve as a better component for enhancing the strength of the resulting composite gels.

**Figure 5:**
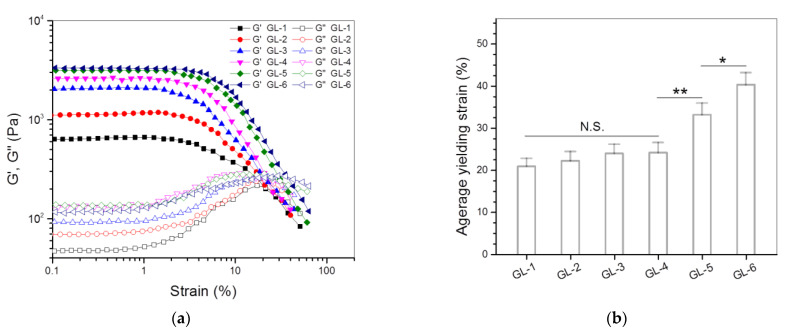
Strain sweep spectra (**a**) and average yielding strain (**b**) for gels illustrated in Table 2 (*, *p* < 0.05; **, *p* < 0.01; N.S., not significant).

The strain dependency of G′ and G″ is often used as an indicator to assess the elasticity of hydrogels [26,28]. In most cases, the strain sweep spectrum of a hydrogel has a crossover point at which G′ is equal to G″, commonly called the yielding strain. Starting from the yielding strain, the increasing strain of the gel will not be in phase with the applied stress, and G′ of the gel will drop down rapidly, which connotes the occurrence of disruption of the 3D network in the gel [26,28]. Figure 5 presents several plots of G′ and G″ versus the strain for different gels illustrated in Table 2. GL-1, GL-2, GL-3 and GL-4 gels were seen to have yielding strains of around 20% or slightly higher without significant differences, suggesting that they are inelastic. GL-5 and GL-6 gels showed significantly larger yielding strains than other gels, manifesting that these gels gain certain elasticity.

As shown in Table 2, GL-4, GL-5 and GL-6 gels were built in the same proportions, but they differed from each other in the ABG NPs incorporated. The bar graphs in Figure 5b propose that the ABG NPs with a longer spacer length can endow the resulting composite gels with significantly improved elasticity.

### 3.3. Dual-Crosslinked Hydrogels

In general, an injectable gel needs a suitable gelation time so that it has good fluidity before injection and, on the other hand, is able to solidify into a self-supporting object within a rational time interval after injection [30]. It is known that GN-involved crosslinking reactions are time-consuming and the gelation time for GN-crosslinked CH hydrogels is usually long [31,32]. In the present instance, the prepared solutions with their formulations shown in Table 2 were estimated to take more than 2 h at 37 °C to become nonflowing, and thereafter, they also needed a long time to form well-solidified gels. The long gelation time for the GN-only crosslinked gels makes them unsuitable for certain in situ gelling applications where fast gelatinization is needed. In addition, the stronger single-crosslinked gels, namely GL-5 and GL-6, only have their G′ values close to 3kPa, which still requires a significant improvement in strength if they are intended for use in bone repair. Considering that GL-6 gel is similar to GL-5 gel in G′ value (Figure 4c) but shows a significantly larger yielding strain (Figure 5b) than GL-5 gel, the GL-6 gel was thus selected for the preparation and subsequent investigation of dual-crosslinked gels.

**Figure 6:**
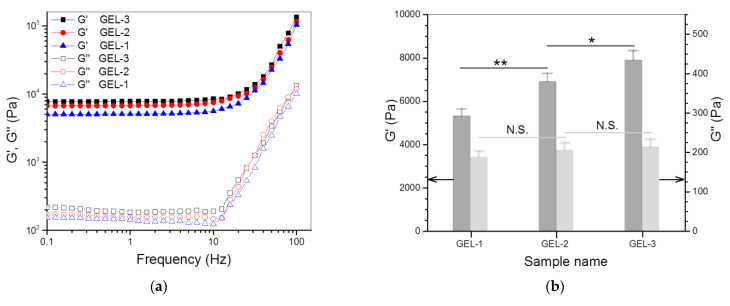

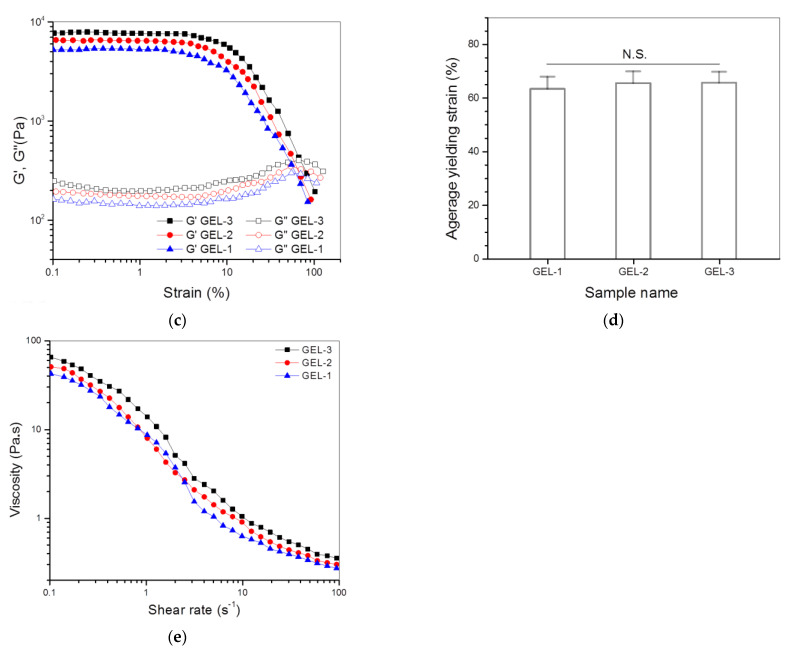
Frequency-dependent functions (**a**) of G′ and G″, average values (**b**) of G′ and G″ at 1 Hz, variations (**c**) in G′ and G″ versus strain, average yielding strain (**d**) and shear rate dependency (**e**) of viscosity (C, 25 °C) for gels illustrated in Table 3 (*, *p* < 0.05; **, *p* < 0.01; N.S., not significant).

PEGDE was employed as another crosslinker due to the high reactivity of the diepoxy groups in PEGDE toward amino and hydroxyl groups [33,34], which could yield multiple networks in the resulting gels when used together with GN and, in turn, impart the heightened strength and enlarged elasticity to the resulting gels. To ensure the adequate safety of composite gels, in the present study, the PEGDE dosage was controlled to a level of 0.03 *w*/*v*% or lower. The compositional proportions, gelation time and some rheological measurement results for the dual-crosslinked gels are provided in Table 3 and Figure 6, respectively. Data in Table 3 reveal that the gelation time for these gels was measured to be from a few minutes to about 10 min with significant dependence on the amount of PEGDE applied, signifying that the gelatinization of the composite solutions was accelerated by PEGDE-involved crosslinking and the gelation rate of the gels can also be tuned by the amount of PEGDE applied.

Figure 6a,b explicate the frequency sweep spectra of G′ and G″ for GEL-1, GEL-2 and GEL-3 gels, and their average values of G′ and G″ at 1 Hz, respectively. These gels showed their G′ of around 5.3, 6.9 and 7.8 kPa, respectively; much greater than that of their counterpart, GE-6 gel. In particular, the G′ value of GEL-2 and GEL-3 gels was more than twice that of GE-6 gel. In addition, the G′/G″ ratios of GEL-2 and GEL-3 gels reached about 33.7 and 37.1, respectively, which were considerably larger than that for GE-6 gel. These results demonstrate that GEL-2 and GEL-3 gels behave as strong gels given their large G′ and high G′/G″ ratios. Figure 6c shows the functions of G′ and G″ versus strain for GEL-1, GEL-2 and GEL-3 gels, and the measured average yielding strains for them are plotted in Figure 6d. The curves and bar graphs symbolize that GEL-1, GEL-2 and GEL-3 gels had similar yielding strains without significant difference, and their yielding strain was notably higher than that of their counterpart, GE-6 gel, providing the evidence that these gels have been further improved in elasticity when compared to GE-6 gel.

It is known that GN is a kind of amino-specific crosslinker [19]. In the single-crosslinked ABG/GCH composite gels, GN can react with amino groups that only belong to GCH chains to build a GCH alone constructed network, and at the same time, it is also able to crosslink amino groups that respectively belong to GCH and ABG NPs to build another network consisting of ABG NPs and GCH. Such built networks would thus enable ABG/GCH composite gels to have certain strength and elasticity with significant dependence on the spacer length of the ABG NPs employed (Figure 4 and Figure 5). Unlike GN, a small molecule crosslinker, PEGDE is a kind of chain-like oligomer with a molecular weight larger than 2 kDa (M_n_ for the PEG segment in PEGDE: 2kDa), and its epoxy groups are capable of reacting with amino or hydroxyl groups. Accordingly, in the case of dual-crosslinked ABG/GCH composite gels, in addition to the presence of the mentioned networks built by the GN-involved linkages, PEGDE would also contribute to the construction of some other networks. The diepoxy groups situated at the two ends of PEGDE molecules can not only react with amino groups belonging to either GCH molecules or ABG NPs, but also react with the hydroxyl groups located at the side chains in GCH molecules. As a consequence, PEGDE-correlated linkages would additionally result in the formation of at least two networks in the ABG/GCH composite gels. Moreover, the PEGDE-bridged linkages in these networks would have certain extensibility since the applied PEGDE molecules have a linear structure and the PEG segment in the PEGDE molecules will exist inside dual-crosslinked ABG/GCH composite gels in the form of random curls, and these PEG curls will unbend during the gel strain. It can also be inferred that the established multiple networks inside dual-crosslinked ABG/GCH composite gels would be randomly interpenetrated and entangled together. All these mentioned factors will synergistically impart the dual-crosslinked ABG/GCH composite gels with significantly enhanced strength and elasticity in comparison to the single-crosslinked composite gels, as evidenced by the results shown in Figure 6.

The degree of crosslinking is known to impose important effects on the structures and properties of hydrogel [26], and, in particular, it is closely correlated to the mechanical performance and swelling behavior of the gels [26,35,36]. The equilibrium swelling measurement and uniaxial compression test are commonly used approaches for estimating the degree of crosslinking of hydrogels [35,36], and the solid state nuclear magnetic resonance spectroscopy has also been shown to be effective for determining the degree of crosslinking of certain hydrogels [37,38]. Based on the Flory theory while assuming a Poisson ratio of 0.5 for the rubber-like hydrogel, the shear modulus (G) of an ideal rubber-like hydrogel can be expressed as follows with a good approximation [39]

(1) $$G=\nu \mathrm{RT}\phi^{1/3}\frac{\bar{r_{0}^{2}}}{\bar{r_{f}^{2}}}$$

where ν is the degree of crosslinking, R is the gas constant, T is the absolute temperature, $\bar{r_{0}^{2}}/\bar{r_{f}^{2}}$ is the front factor and ϕ is the volume fraction of the polymer in the swollen hydrogel. By assigning a value of unity to the front factor, Equation (1) can be simplified into the following formula [40]

(2) $$\left|G^{*}\right|=\nu \mathrm{RT}\phi^{1/3}$$

The degree of crosslinking for the gels illustrated in Table 3 was calculated using Equation (2) and such approximately obtained results are also provided in Table 3. It can be observed that there were significant differences in the degree of crosslinking among GEL-1, GEL-2 and GEL-3 gels, and the degree of crosslinking increased with the amount of PEGDE applied. By comparing the data in Table 3 with those shown in Figure 6b, it can be seen that the crosslinking degree of the gels changed in a manner similar to the variation trend of their G′, signifying that the gels can be strengthened by raising their degree of crosslinking. This is understandable because PEGDE-bridged linkages will increase as the amount of PEGDE applied rises, which would thus lead to augmented network entanglement in the resulting gel, thereby enhancing its strength. Despite the differences in the degree of crosslinking among GEL-1, GEL-2 and GEL-3 gels, they had similar average yielding strains (Figure 6d), implying that the degree of crosslinking has an insignificant effect on their elasticity. One possible reason for the insignificant effect of the crosslinking degree may be ascribed to the following tentative interactions. In general, an increase in the gel strength will result in a corresponding rise in its deformation stress. In the present instance, for the gel having a higher degree of crosslinking, its enhanced resistant force against the deformation could be counteracted by its increased deformation stresses, resulting in a similar yield strain when compared to the gel having a relatively lower degree of crosslinking.

Taking into account the injection applicability of gels, GEL-1, GEL-2 and GEL-3 solutions were tested for their viscosity versus shear rate, and the results are elucidated in Figure 6e. In the lower shear rate range with an upper limit of around 10 s^−1^, these composite solutions were viscous with similarity in their viscosity, and their viscosity became notably low once the applied shear rate was higher than 10 s^−1^, indicating that they have shear-thinning features. Considering that the injection of composite solutions is usually conducted at ambient temperature, the results in Figure 6e account for their well-defined injectability.

### 3.4. Cell Growth and Analysis

**Figure 7:**
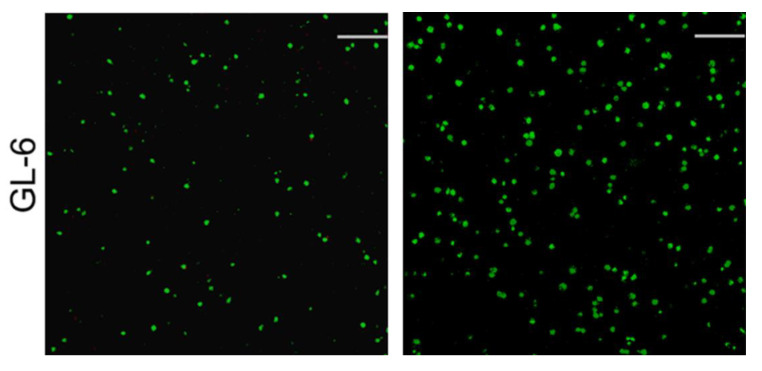

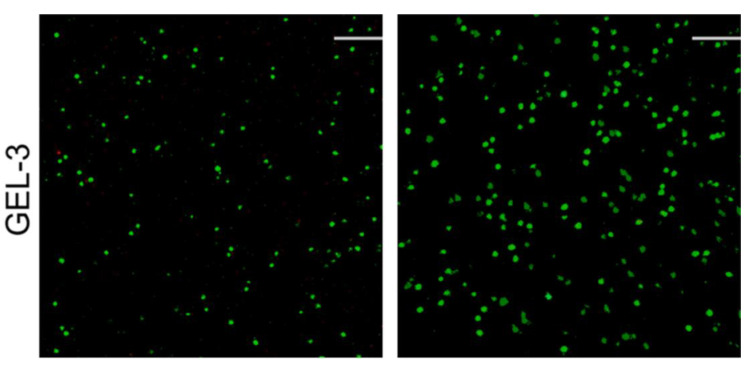
Confocal images for stained MC3T3-E1cells that were seeded GL-6 and GEL-3 gels (green: viable cells; red: dead cells; scale bar: 100 μm; culture time: 3 days (left column) and 7 days (right column)).

The GEL-3 gel had its G′ significantly higher than GEL-1 and GEL-2 gels, and it was similar to GL-6 gel with a difference in the applied crosslinkers. GL-6 and GEL-3 gels were thus selected for the in vitro evaluation. Figure 7 presents representative fluorescence images for the stained MC3T3-E1 cells that were seeded in GL-6 and GEL-3 gels and cultured for various periods up to 7 days. Images in both columns display that very few dead cells were detected from these gels after the culture of cell–gel constructs for 3 (left column) or 7 (right column) days, and the cell density in the images matching with 7-day culture was markedly higher than that corresponding to 3-day culture. These images confirm that the seeded MC3T3-E1 cells had high viability, and GL-6 and GEL-3 gels had similar capabilities to support the growth of seeded cells.

**Figure 8:**
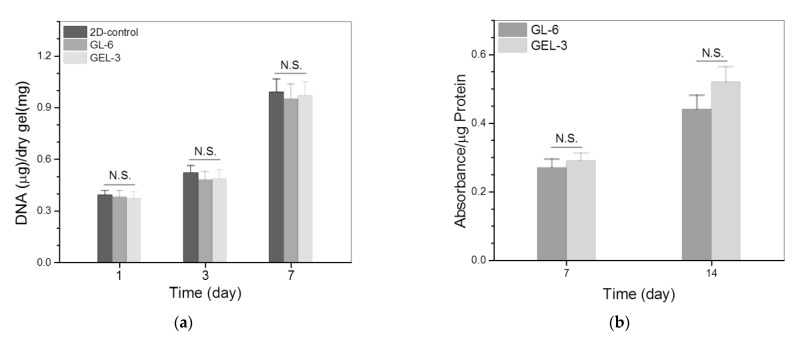

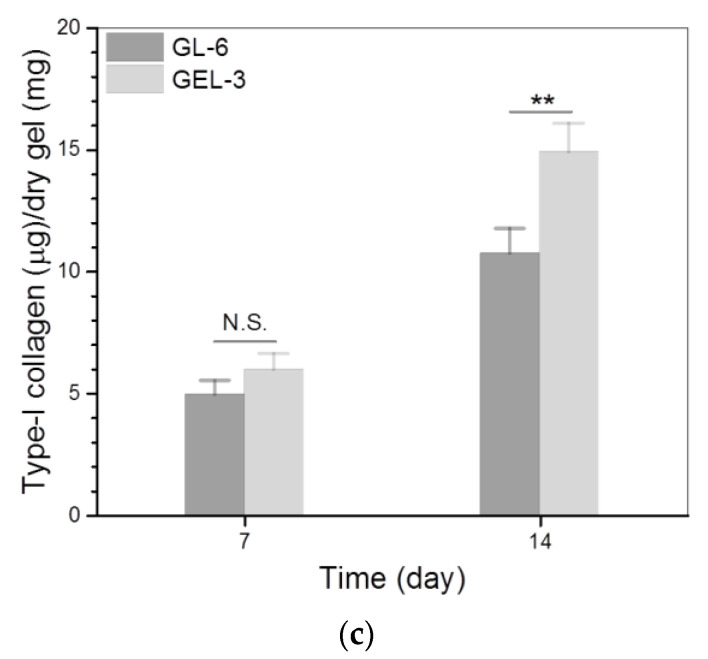
Proliferation (**a**), ALP activity (**b**) and type-I collagen deposition (**c**) of MC3T3-E1 cells growing inside gels (**, *p* < 0.01; N.S., not significant).

Figure 8a delineates the results for the cell proliferation in different gel matrices. It can be observed that the growth of seeded cells can be roughly divided into two phases: they grew slowly from days 1 to 3 and then grew faster from days 3 to 7. The slow cell growth in the first phase is due to the cell attachment as well as subsequent population recovery, and the fast cell growth in the second phase can be ascribed to the occurrence of cell proliferation. There were no significant differences in the detected DNA amounts among these groups during one-week culture, revealing that they have similar abilities to support the proliferation of seeded MC3T3-E1 cells. Considering that the GL-6 and GEL-3 gels are different in terms of the crosslinkers applied, the results shown in Figure 7 and Figure 8a suggest that the applied amount of GN and the combined amount of GN and PEGDE are all within the safe dose range.

ALP activity is a commonly used indicator for evaluating the osteogenic development of cells in the early stage [41,42]. The cell–gel constructs were thus detected to assess the ALP activity of seeded cells, and relevant results are graphed in Figure 8b. The bar graphs exhibit that there was no significant difference in the ALP activity for the cells seeded in the GL-6 or GEL-3 gels after 7-day culture. After being extensively cultured for a period of up to 14 days, the ALP activity detected from these gels remarkably increased without significant difference. As shown in Table 2 and Table 3, the GL-6 and GEL-3 gels differed from each other in the crosslinkers applied and, in turn, in their strength and elasticity. The results depicted in Figure 8b indicate that the mentioned differences between the GL-6 and GEL-3 gels impose an insignificant impact on the ALP activity of the cells seeded in these gels.

The BG NPs composed of CaO and SiO_2_ are known to be able to release Si and Ca ions during their dissolution, and these two kinds of ions are demonstrated to have osteogenic activity [17,18]. In the present study, ABG-3 NPs were made from BG NPs by surface chemical modification; hence, they would also be able to release Si and Ca ions during the dissolution and, accordingly, endue the resulting GL-6 and GEL-3 gels with certain osteogenic activity, denoted by the increasing ALP levels. The insignificant difference in the ALP activity detected from the GL-6 and GEL-3 gels can be ascribed to the fact that the GL-6 and GEL-3 gels are incorporated with the same amount of ABG-3 NPs.

Type-I collagen synthesized by the osteoblast-like cells is another important indication correlated to the osteogenic development of the cells [43]. The amount of type-I collagen in the cell–gel constructs was thus measured to figure out the effect of the gels on the type-I collagen deposition, and the obtained data are plotted in Figure 8c. It is shown that the type-I collagen amounts deposited in the GL-6 and GEL-3 gels were similar without significant difference after 7-day culture. After being seeded in these gels, MC3T3-E1 cells need to go through the process of adhesion, restorative growth and the subsequent proliferation, and a 7-day growth period could be too short for them to secrete a large amount of type-I collagen, leading to the insignificant difference in their type-I collagen deposition. Type-I collagen deposition was noticed to increase at varied rates for GL-6 and GEL-3 gels as the culture time advanced from day 7 to day 14, and the deposited amount of type-I collagen for GEL-3 gel was remarkably higher than that for GL-6 gel on day 14. Data presented in Table 1 and Figure 4, Figure 5 and Figure 6 exhibit that GEL-3 gel differed from GL-6 gel in the ingredient of PEGDE as well as in the strength and elasticity. Hence, the significantly higher type-I collagen deposition in GEL-3 gel should be majorly attributed to its higher strength and elasticity when compared to GL-6 gel.

Both CH and BG NPs have been widely used in bone tissue engineering since the former can act as bone affinitive material to well support cell growth and ECM synthesis, and the latter has a demonstrated osteogenic activity [11,44,45,46]. Nevertheless, A CH-based hydrogel intended for use in bone repair still needs improvement to increase its strength and make it elastic enough because a strong and elastic microenvironment is required for the cells implanted or migrated from the host tissue to repair the injured bone tissue. In the present study, GCH, a water-soluble derivative of CH, and ABG NPs, a modified product of BG NPs, were employed as two major components for the preparation of composite hydrogels, and the optimally achieved dual-crosslinked ABG/GCH gels were demonstrated to be strong and elastic with the abilities to support the growth of seeded MC3T3-E1 cells and the synthesis of matrix components. These results suggest that these composite hydrogels have the potential for applications in bone tissue engineering.

## 4. Conclusions

Three kinds of mesoporous ABG NPs with similar amounts of surface amino groups but varied spacer lengths were successfully synthesized via newly devised synthetic routes. By combining ABG NPs with GCH while using GN as a single crosslinker, the constructed ABG/GCH gels had their strength and elasticity with certain dependence on the spacer length of ABG NPs. The ABG NPs with a longer spacer length would endow the resulting ABG/GCH gels with higher strength or larger elasticity when compared to those ABG NPs having a shorter spacer length. By employing GN and PEGDE as dual crosslinkers, the dual-crosslinked ABG/GCH gels would be remarkably improved in their strength and elasticity while showing tunable gelation time. Some optimally dual-crosslinked ABG/GCH gels were capable of supporting the growth of the seeded osteoblast-like cells as well as the matrix deposition. The higher strength and larger elasticity of the gels were found to be conducive to the synthesis of type-I collagen. Results demonstrate that this new type of dual-crosslinked hydrogel has potential for applications in bone repair.

## Figures and Tables

**Table 1:** Parameters for different kinds of nanoparticles.

Sample Name	Surface Area (m^2^/g)	Pore Volume (mL/g)	Pore Size (nm)	ζ-Potential (mV)	Particle Size (nm)	Content of Amino Groups (mmol/g)
BG	794.4 ± 63.2	1.03 ± 0.09	4.94 ± 0.21	−12.8 ± 0.81	251.6 ± 10.8	−
ABG-1	462.1 ± 31.7	0.81 ± 0.06	4.06 ± 0.17	27.3 ± 1.27	279.9 ± 17.5	0.426 ± 0.031
ABG-2	445.4 ± 27.4	0.74 ± 0.07	3.74 ± 0.13	26.7 ± 1.32	298.1 ± 20.7	0.407 ± 0.046
ABG-3	417.8 ± 23.1	0.68 ± 0.05	3.38 ± 0.15	26.4 ± 1.49	314.4 ± 21.1	0.386 ± 0.042

**Table 2:** Parameters for single-crosslinked hydrogels.

Sample Name	GCH (*w*/*v*%)	ABG-1 (*w*/*v*%)	ABG-2 (*w*/*v*%)	ABG-3 (*w*/*v*%)	GN (*w*/*v*%)
GL-1 ^(a)^	2.5	−	−	−	0.2
GL-2	2.5	1	−	−	0.2
GL-3	2.5	1.5	−	−	0.2
GL-4	2.5	2.0	−	−	0.2
GL-5	2.5	−	2.0	−	0.2
GL-6	2.5	−	−	2.0	0.2

(a) This gel was used as control.

**Table 3:** Parameters for dual-crosslinked hydrogels.

Sample Name	GCH (*w*/*v*%)	ABG-3 (*w*/*v*%)	GN (*w*/*v*%)	PEGDE (*w*/*v*%)	Gelation Time at 37 °C (s) ^(a)^	Degree of Crosslinking (×10^−5^ mol/cm^3^) ^(b)^	ϕ
GEL-1	2.5	2.0	0.2	0.01	630 ± 24.4	0.902 (±0.063)	0.0128
GEL-2	2.5	2.0	0.2	0.02	285 ± 17.3	1.122 (±0.051) *	0.0147
GEL-3	2.5	2.0	0.2	0.03	207.5 ± 20.6	1.23 (±0.047) ^#^	0.0161

(a) The gelation time was determined by inverting the vials every 30 s. (b) *, *p* < 0.05 compared to GEL-1; #, *p* < 0.05 compared to GEL-2.

## Data Availability

The data presented in this study are available on request from the corresponding author.
